# Inflammation and neurodegeneration: chronicity matters

**DOI:** 10.18632/aging.101704

**Published:** 2018-12-16

**Authors:** Keenan A. Walker

**Affiliations:** 1 Department of Neurology, Johns Hopkins University, Baltimore, MD 21287, USA

**Keywords:** inflammation, dementia, neurodegeneration, white matter, Alzheimer’s disease

Although it has become clear over the last several decades that inflammation plays a role in the pathogenesis of Alzheimer’s disease and other forms of dementia, the precise nature and temporal characteristics of the neurodegeneration-inflammation relationship have remained largely unknown. Several lines of research have identified inflammation, both within the brain and within the periphery, as a potential driver of neurodegenerative brain changes and cognitive decline. *Chronic low-grade inflammation,* in particular, has received considerable attention, as translational studies suggest that it may play a causal role in dementia, late-life cognitive decline, and a number of other age-related phenotypes [1]. Inflammation is a ubiquitous feature of aging that can occur secondary to a number of physiological and disease states, including obesity, cardiovascular disease, and infection. Especially among older adults, chronic inflammation can also occur independent of disease as a result of several age-related physiological changes, such as cellular senescence and the accumulation of cellular debris. This age-related predisposition for chronic inflammation has been termed *inflammaging* [1].

**Figure 1:**
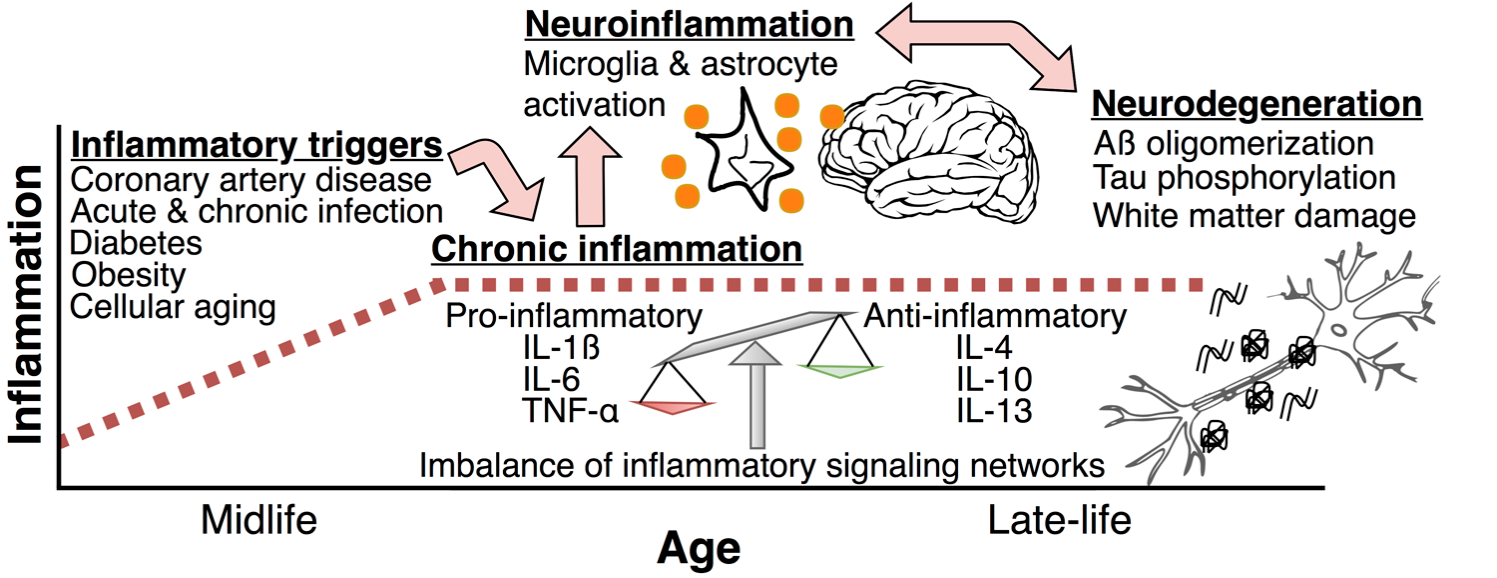
This figure describes the hypothesized relationship between systemic inflammation, neuroinflammation, and neurodegeneration. Inflammatory triggers, such as infection, chronic disease, and cellular aging can occur before or during middle adulthood. These events lead to increased pro-inflammatory signaling within the periphery, which can result in long-term perturbations in the balance between pro-inflammatory and anti-inflammatory networks. For some, these changes can lead to a state of chronic systemic inflammation that persists into older adulthood. By activating microglia and astrocytes, systemic inflammation can initiate or perpetuate a cytokine- and complement-mediated inflammatory response within the brain (i.e. neuroinflammation). Neuroinflammation has been shown to promote pathogenic brain changes, such as increased amyloid precursor protein processing, beta-amyloid oligomerization, tau phosphorylation, and small vessel disease. IL-1ß = interleukin 1 beta; IL-4 = interleukin 4; IL-6 = interleukin 6; IL-10 = interleukin 10; IL-13 = interleukin 13; TNF-α = tumor necrosis factor alpha.

Systemic inflammatory signaling, which is characterized by the proliferation of pro-inflammatory mediators throughout the body, can directly influence functioning within the central nervous system and cause changes in cognition and behavior. The most common example of this neural-immune interaction is the sickness behavior (fatigue, loss of appetite, apathy) which occurs as a result of infections such as the common cold. Inflammatory mediators, such as cytokines, can promote an inflammatory state within the central nervous system by (1) crossing the blood brain barrier or entering through the brain’s circumventricular organs, (2) communicating with the central nervous system directly via afferent vagal nerve signaling, and (3) signaling indirectly via the cerebral endothelium [2]. Through these pathways, systemic inflammation can both incite and perpetuate a pro-inflammatory response within the brain (i.e., neuroinflammation) and promote a number of other molecular changes, such as beta-amyloid (Aβ) oligomerization and tau phosphorylation, which ultimately lead to pathogenic changes in brain structure and function (see Figure 1) [3]. Accordingly, these neural-immune pathways have been implicated in a number of neuropsychiatric conditions, including delirium, depression, and Alzheimer’s disease.

Although evidence from animal models indicates that chronic inflammation can perpetuate, or even initiate, neurodegenerative changes, this hypothesis has been challenging to examine in human studies. This is largely due to a lack of longitudinal data on inflammatory biomarkers in cohort studies which examine neurocognitive outcomes in older adults. In a recently published study of participants from the Atherosclerosis Risk in Communities (ARIC) Cohort, we were able to clarify the relationship between long-term (21-year) patterns of systemic inflammation and late-life neurodegenerative changes [4]. The ARIC Study, which has followed a large community-based cohort for approximately 30 years since middle adulthood, provided a unique opportunity to relate patterns of systemic inflammation (measured using blood C-reactive protein [CRP] levels) to MRI-defined neurodegeneration. In this study, we found that individuals who both demonstrated elevated inflammation before or during middle adulthood and maintained high levels of inflammation over the subsequent two decades had greater white matter hyperintensity volume and reduced white matter microstructural integrity as older adults, compared to those who maintained low levels of inflammation. These results suggest that chronic systemic inflammation may contribute to the development of cerebral white matter abnormalities: an indicator of tissue damage and axonal pathology, and a common feature of dementia. These findings are consistent with other recently published results, which indicate that individuals with elevated systemic inflammation during midlife [5] and those who experience acute inflammatory triggers (e.g., major infection) [6] have smaller brain volumes as older adults, particularly in brain regions vulnerable to Alzheimer’s disease.

Together, these findings support the idea that systemic inflammation can initiate or perpetuate neurodegenerative brain changes which underlie cognitive impairment and dementia. To the best of our knowledge, these results are among the first to demonstrate that individuals with persistent or chronic elevations in inflammation over many years are at greatest risk for neurodegenerative white matter changes as older adults. While we suspect that long-term systemic inflammation may contribute to neurodegenerative pathology by signaling, and thus perpetuating, the neuroinflammatory response (as illustrated in Figure 1) [7], this has not yet been demonstrated in humans. Future longitudinal studies will be needed to determine how long-term patterns of systemic inflammation affect inflammatory signaling within the brain. Systemic inflammation and neuroinflammation represent potential treatment targets for slowing the progression of neurodegenerative disease. However, an improved understanding of the dynamic longitudinal relationship between inflammation and components of the neurodegenerative process will likely be needed before successful targeted interventions can be developed.
